# No gadolinium K‐edge detected on the first clinical photon counting computed tomography scanner: Response to Dr. Liu and Dr. Noel

**DOI:** 10.1002/acm2.14443

**Published:** 2024-06-18

**Authors:** Erik Baubeta, Eva Laurin Gadsböll, Leon Will, Fredrik Holmquist, Marie‐Louise Aurumskjöld

**Affiliations:** ^1^ Department of Imaging and Functional Medicine Skåne University Hospital Lund Sweden; ^2^ Department of Translational Medicine, Diagnostic Radiology Lund University Malmö Sweden; ^3^ Department of Clinical Sciences, Diagnostic Radiology Lund University Lund Sweden; ^4^ Medical Radiation Physics, Dept. of Clinical Sciences Malmö, Skåne University Hospital Lund University Malmö Sweden

Dear Editor,

We appreciate the relevant and insightful comments on our publication entitled “No gadolinium K‐edge detected on the first clinical photon‐counting computed tomography scanner.” While we largely share the raised objections in a broader sense, the aim of our study was to assess the capabilities of the first generation of clinically available photon‐counting computed tomography (PCCT). This focus stems from the significant potential advantage of PCCT technology in differentiating between various materials, a feature prominently highlighted by manufacturers in their marketing.

PCCT could theoretically sort incoming photons into different energy bins based on their measured energies. This energy discrimination is critical for k‐edge imaging, as it allows for the detection of photons just above and below the k‐edge energy. Special reconstruction techniques can enhance the visibility of the k‐edge by emphasizing the energy bins around the k‐edge. This improves the contrast and specificity of the imaging. As the correspondents point out, more sophisticated multi‐material decomposition techniques, employing data from the detector to specific bins, would have been preferable, but are not available at the first clinical generation PCCT. Therefore, we have tried to optimize the use of the machine's features, including testing different contrast concentrations and optimizing the radiation dose, trying to work around these shortcomings. Higher concentrations of Gd could enhance the K‐edge signal, which has been used in several preclinical animal studies, but would imply toxic levels in humans. Additionally, we reached out to the manufacturer of the machine for assistance in improving the outcome but did not receive further support.

Therefore, our current findings suggest that gadolinium may not be effective as a contrast agent with the currently available clinical PCCT technology. We believe this conclusion is relevant to the broader clinical radiology community, providing realistic expectations of today's technology and guidance on its implementation in current healthcare and research practices. Nevertheless, we remain optimistic that future advancements in PCCT technology may demonstrate greater efficacy in this regard. However, further product development is necessary before these improvements can be realized in a clinical setting.

In contrast to the correspondents, we believe that our results actually strengthen, rather than weaken, the incentives for further research and implementations of advancements in PCCT technology. Theoretical calculations and preclinical trials have shown how PCCT can fundamentally change our use of CT. Even though the first generation of PCCT may not unleash the full potential of the technology, we certainly do not consider the race lost. Instead, we will continue to strive to optimize and develop PCCT technology for tomorrow's diagnostics.

## AUTHOR CONTRIBUTIONS

*Conceptualization, Sample preparation and collection, writing and drafting the manuscript*: Erik Baubeta. *Sample preparation and collection, drafting, finalizing and approval of the manuscript*: Eva Laurin Gadsböll. *Contributed to sample preparation, drafting, finalizing and approval of the manuscript*: Leon Will. *Sample preparation, drafting manuscript and final approval*: Fredrik Holmquist. *Conceptualization, sample preparation and collection, drafting, finalizing and approval of the manuscript*: Marie‐Louise Aurumskjöld.

